# Supplementary material for: Structural mechanism of signal transduction in a phytochrome histidine kinase
Source: Nat Commun. 2022 Dec 12;13:7673. doi: 10.1038/s41467-022-34893-3 (PMC9744887; doi:10.1038/s41467-022-34893-3)
Supplement: Supplementary file 1 — Supplementary Information [file 41467_2022_34893_MOESM1_ESM.pdf]

Supplementary Information

Structural mechanism of signal transduction in a phytochrome histidine kinase

Weixiao Yuan Wahlgren[1], Elin Claesson[1], Iida Tuure[2], Sergio Trillo-Muyo[3], Szabolcs Bódizs[1], Janne A. Ihalainen[2], Heikki Takala[2,4,*], and Sebastian Westenhoff[1,5,*]

Affiliations:
[1] Department of Chemistry and Molecular Biology, University of Gothenburg; 40530 Gothenburg, Sweden

[2] Nanoscience Center, Department of Biological and Environmental Science, University of Jyvaskyla, 40014 Jyvaskyla, Finland

[3] Department of Medical Biochemistry and Cell Biology, University of Gothenburg; 413 90 Gothenburg, Sweden

[4] Faculty of Medicine, Anatomy, University of Helsinki; 00014 Helsinki, Finland

[5] Department of Chemistry - BMC, Biochemistry, Uppsala University, 75123 Uppsala, Sweden

*Correspondence to: heikki.p.takala@jyu.fi, westenho@chem.gu.se

Supplementary Information

Supplementary Information file include Supplementary Figures 1–8 and Supplementary References.

Supplementary Figure 1: Gel chromatogram, dark reversion, and activity of purified *Dr*BphP-*Dr*RR.

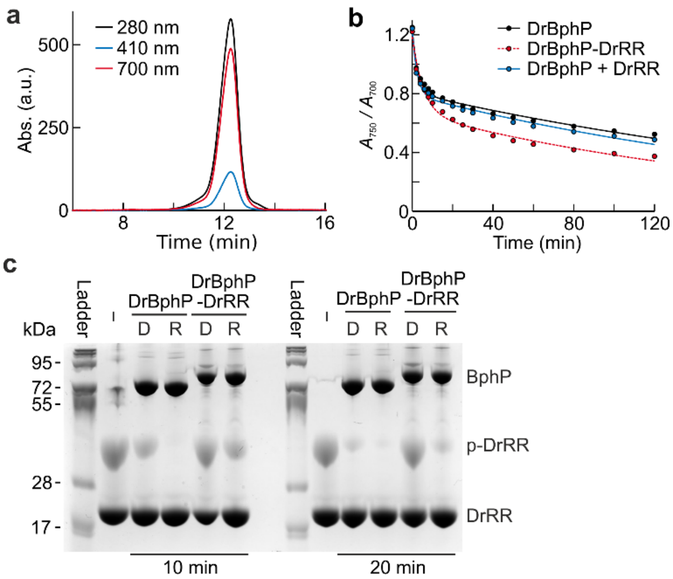

(**a**) Size-exclusion chromatogram of *Dr*BphP-*Dr*RR prior to deposition on the grids for cryo EM, plotted at three phytochrome-specific wavelengths. The chromatogram indicates pure and monodisperse sample. (**b**) Dark reversion of *Dr*BphP-*Dr*RR fusion and *Dr*BphP with added *Dr*RR,[1] conducted as in the main text. External addition of excess *Dr*RR (10 µM) to *Dr*BphP (1 µM) increased the dark reversion rate of the phytochrome. However, this reversion rate was slower than with the fusion, which can be explained by high local concentration of *Dr*RR in the *Dr*BphP binding site of the fusion and by different buffer conditions used in Multamäki et al. 2021.[1] The decay times (with 95% confidence bounds and amplitude) were τ_1 = 2.9 min (2.2 min, 4 min, 35%) and τ_2 = 246 min (208 min, 301 min, 65%) for *Dr*BphP; τ_1 = 5.4 min (4.2 min, 7.7 min, 44%) and τ_2 = 179 min (141 min, 244 min, 56%) for *Dr*BphP-*Dr*RR; and τ_1 = 2.4 min (1.8 min, 3.4 min, 36%) and 216 min (184 min, 260 min, 64%) for *Dr*BphP + *Dr*RR. (**c**) Extended version of the gel shown in Figure 1d. The gel contains the same experiment with two different incubation times (10 min and 20 min). We observe that the amount of remaining p-*Dr*RR depends on the incubation time, but that the relative phosphatase activities of each sample remain approximately the same. For source data, see separate Source Data file.

Supplementary Figure 2: Scheme for reconstruction of the electron density maps in Pr.

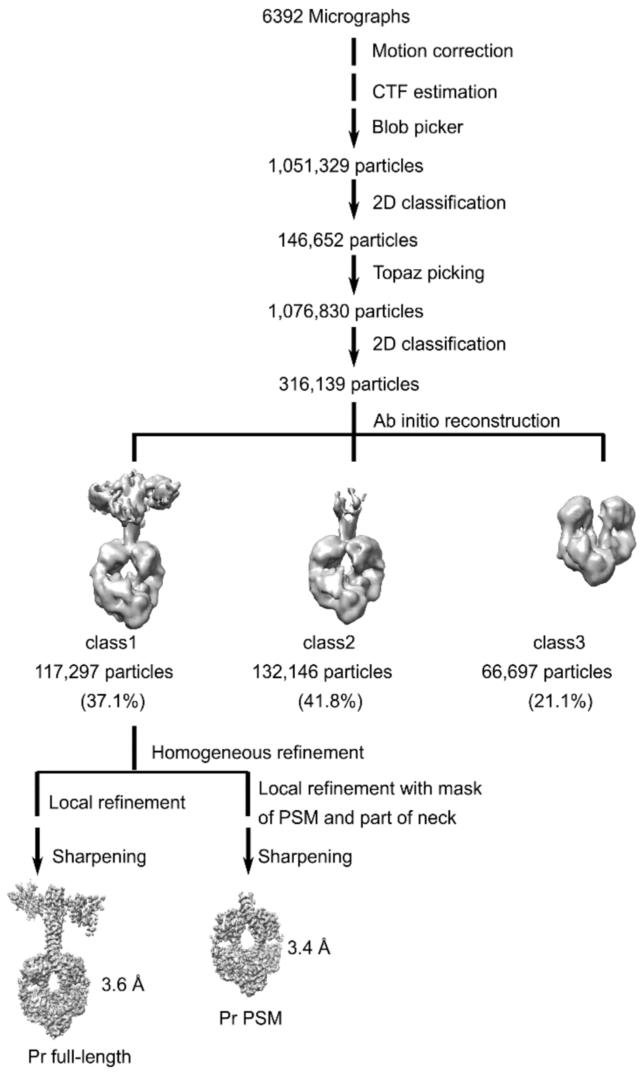

Summary of the reconstruction procedure from single-particle images. After *ab initio* reconstruction, class2 and class3 contain impurities and broken particles and were therefore discarded. The best 3D reconstruction class1 was used for further refinement steps. The final full-length 3D reconstruction of Pr state has an overall resolution of 3.6 Å. The local refinement with a mask of PSM and part of the neck region yields an improved overall resolution of 3.4 Å.

Supplementary Figure 3: Homology models indicate a potential wide conservation of the helical neck and comparison of cryo-EM to crystal structures.

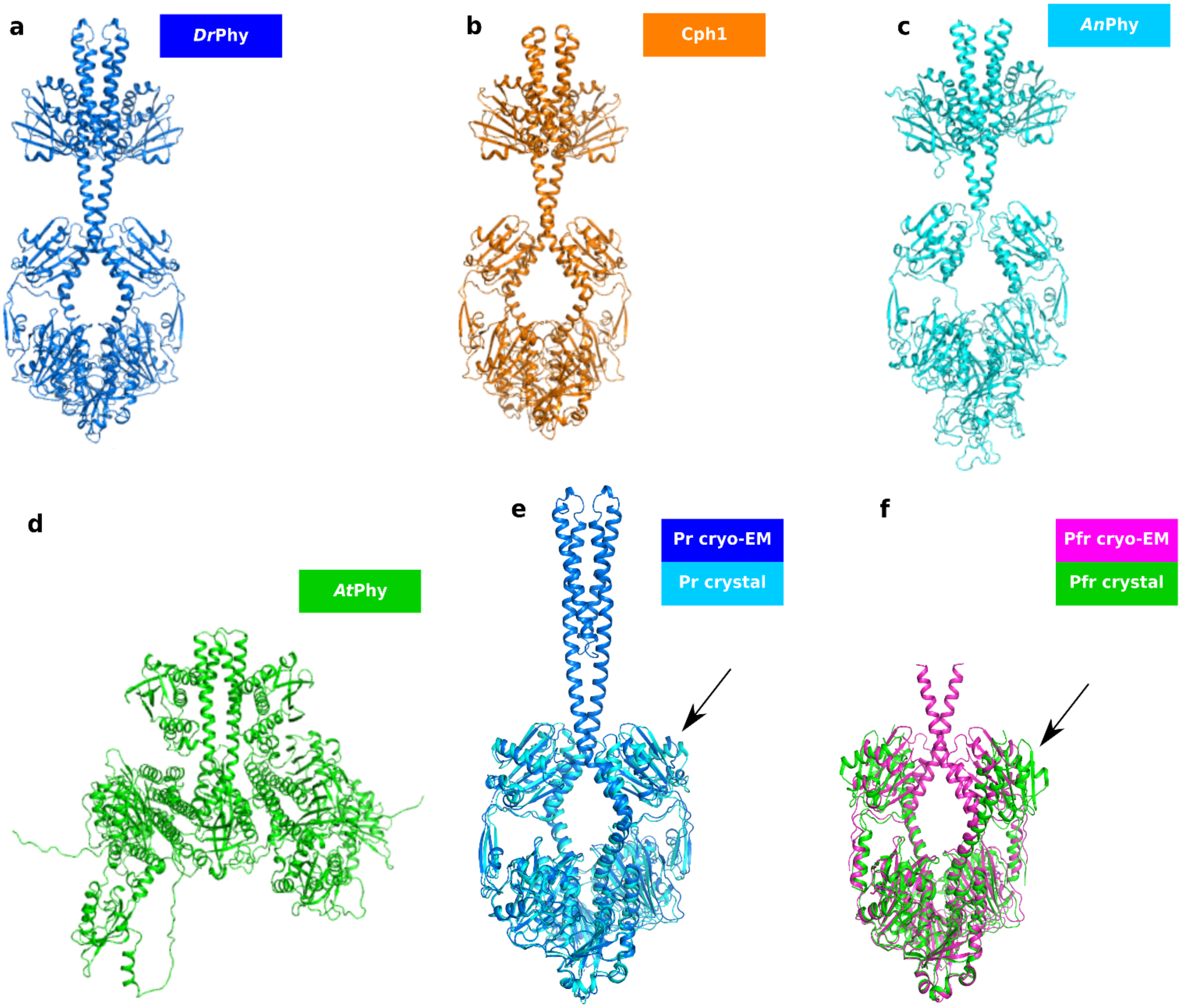

(**a-d**) The "neck" is a conserved feature across the phytochrome superfamily. The reconstructed Pr state of *Dr*BphP (Q9RZA4) (**a**) was compared to SWISS-MODEL homology models of cyanobacterial Cph1 (Q55168) (**b**), Fungal *En*FphA (Q5K039) (**c**), and an AlphaFold model of plant *At*PhyA (P14712) (**d**). All homologues possess a neck region with buried interface (shown with arrows), suggesting that the photoactivation mechanism may be conserved. (**e-f**) Comparison of the crystal structures of the photosensory module of *Dr*BphP (Q9RZA4, pdb codes 4O0P for Pr and 4O01 for Pfr) overlaid with the structures obtained in this work indicate that the Pr crystal structure overlaps well (RMSD 1.330 for residues 22-502), but that the Pfr crystal structure diverges (RMSD 1.416 for residues 22-502). See arrows for largest discrepancies.

Supplementary Figure 4: Scheme for reconstruction of the electron density maps in Pfr.

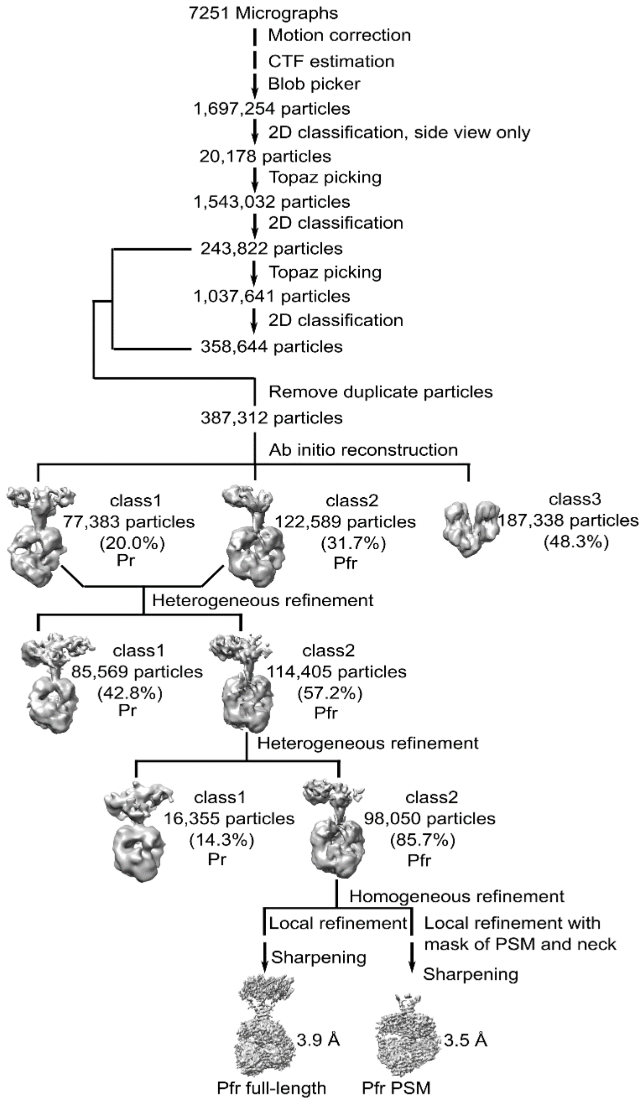

Summary of the reconstruction procedure from single-particle images. After *ab initio* reconstruction, class3 was discarded. It contained incomplete particles and impurities. Class1 was similar to the full-length model of Pr from the experiment in the dark (Supplementary Fig. 2), as it contained densities for a β sheet in the tongue region. Class2 gave a different full-length model compared to class 1 and showed a novel positioning of the output domains and helical densities in the tongue region. We assign this class to Pfr. For further refinement, we first pooled all particles from class1 and class2 for heterogeneous refinement, using class1 and class2 as 3D reference models. This essentially reassigns and realigns particles to the Pr and Pfr classes. A second run of heterogeneous refinement was performed on class 2 to further clean out Pr particles from the Pfr density. The final model of Pfr (3.9 Å resolution) had the hallmarks of a Pfr structure (see main text). By applying a mask over the PSM and part of neck, the Pfr PSM model was refined to 3.5 Å. Approximately 50% of the intact particles (class 1 and 2 after *ab initio* refinement) were in Pfr and 50% in Pr, close to the maximum turnover of the photoreaction of approximately 65%.

Supplementary Figure 5: Assignment of electron densities to CA and REC domains.

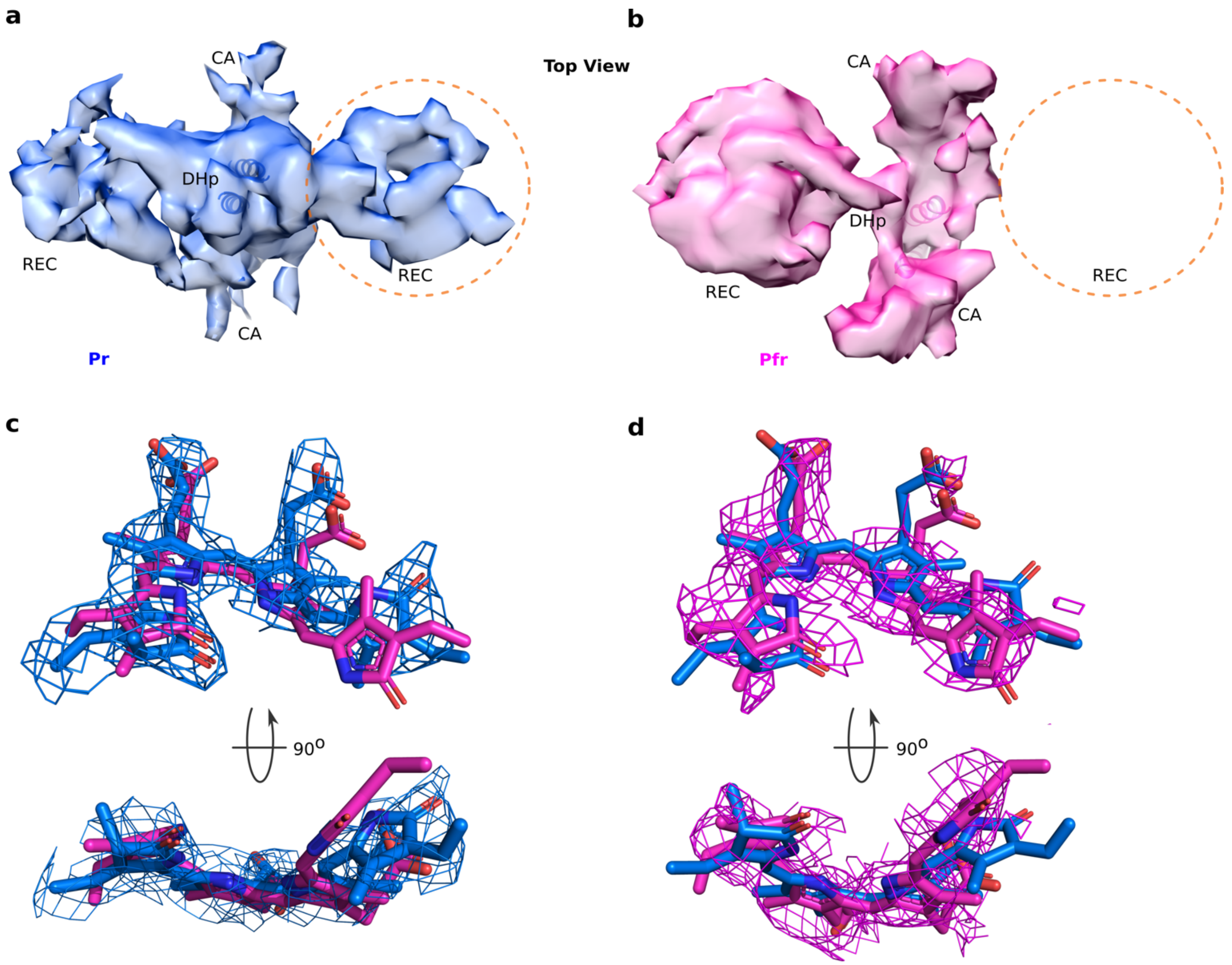

(**a**) Assignment of electron densities in Pr to CA and REC domains. The CA and REC domains are assigned as indicated on the map (blue with 50% transparency) from class 1 of the *ab initio* reconstruction step (Supplementary Fig. 2). The modelled Pr structure (PAS-GAF-PHY-neck, blue) was fit into the density. (**b**) Assignment of electron densities in Pfr to CA and REC domains. The Pfr structures (PAS-GAF-PHY-neck, magenta) is shown with the Pfr electron density (magenta with 50% transparency) from class 2 of the ab-initio reconstruction step (Supplementary Fig. 4). The density of the effector domains is asymmetric. We tentatively assign patches of density to the CA domains of both monomers and the REC domain of one of them as indicated. The neck helices are visualized from the top through the electron densities in both panels a and b. (**c**) Biliverdin in Pfr (magenta) and Pr (blue) superimposed with the electron density in Pr (blue). (**d**) Superimposed biliverdin in Pr (blue) and Pfr (magenta) with the electron density in Pfr (magenta).

Supplementary Figure 6: Local resolution and Fourier Shell Correlation of the maps in Pr.

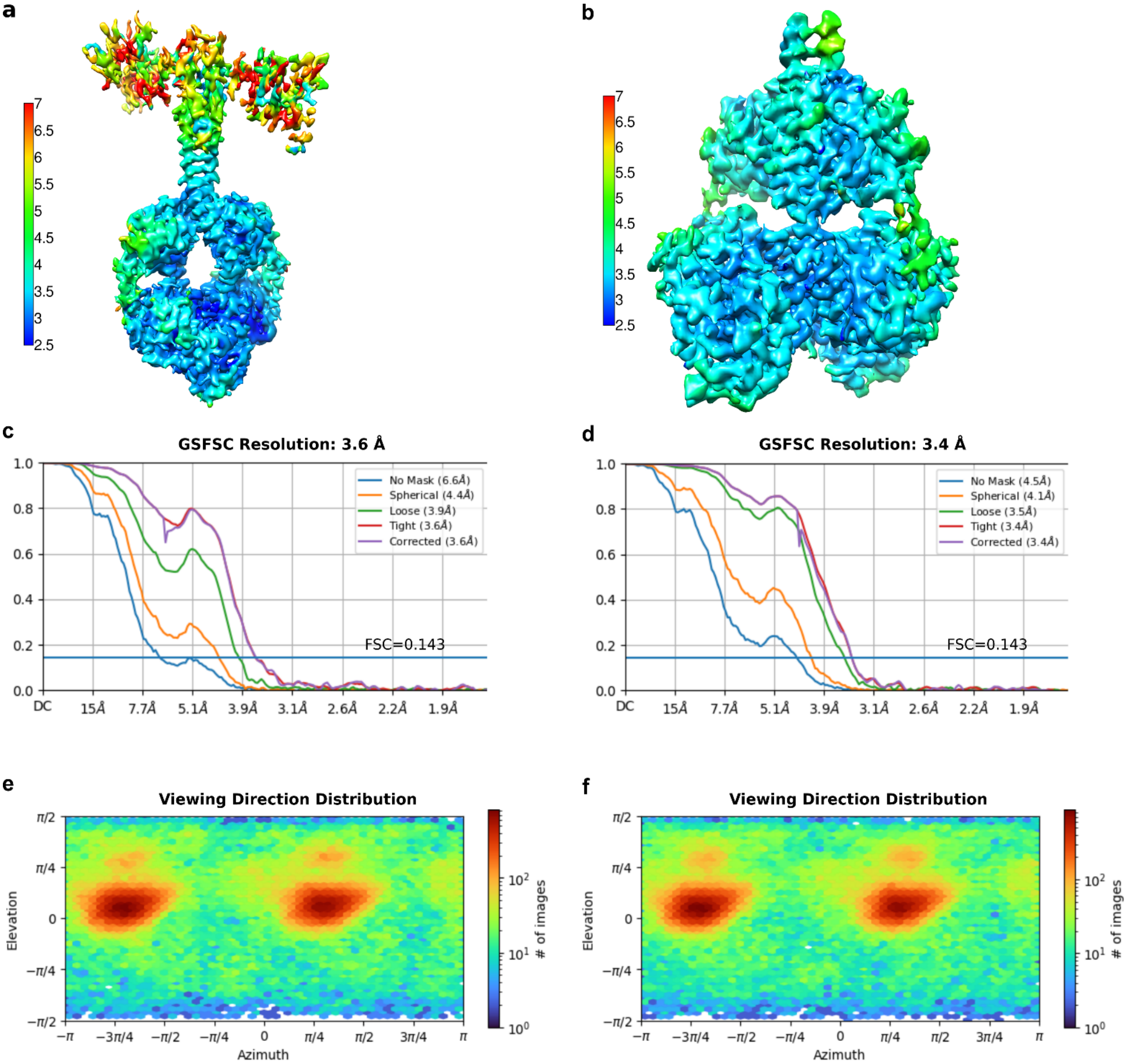

The sharpened cryo-EM densities of the Pr state of (**a**) full-length and (**b**) PSM, colored based on local resolution produced using local resolution estimation in cryoSPARC. Fourier Shell Correlation curves for the cryo EM maps of (**c**) full-length and (**d**) PSM are also shown. The resolution of map corresponds to FSC 0.143. Angular distribution calculated in cryoSPARC for particle projections of (**e**) full-length and (**f**) PSM. The heat maps show number of particles for each viewing angle.

Supplementary Figure 7: Local resolution and Fourier Shell Correlation of the maps in Pfr.

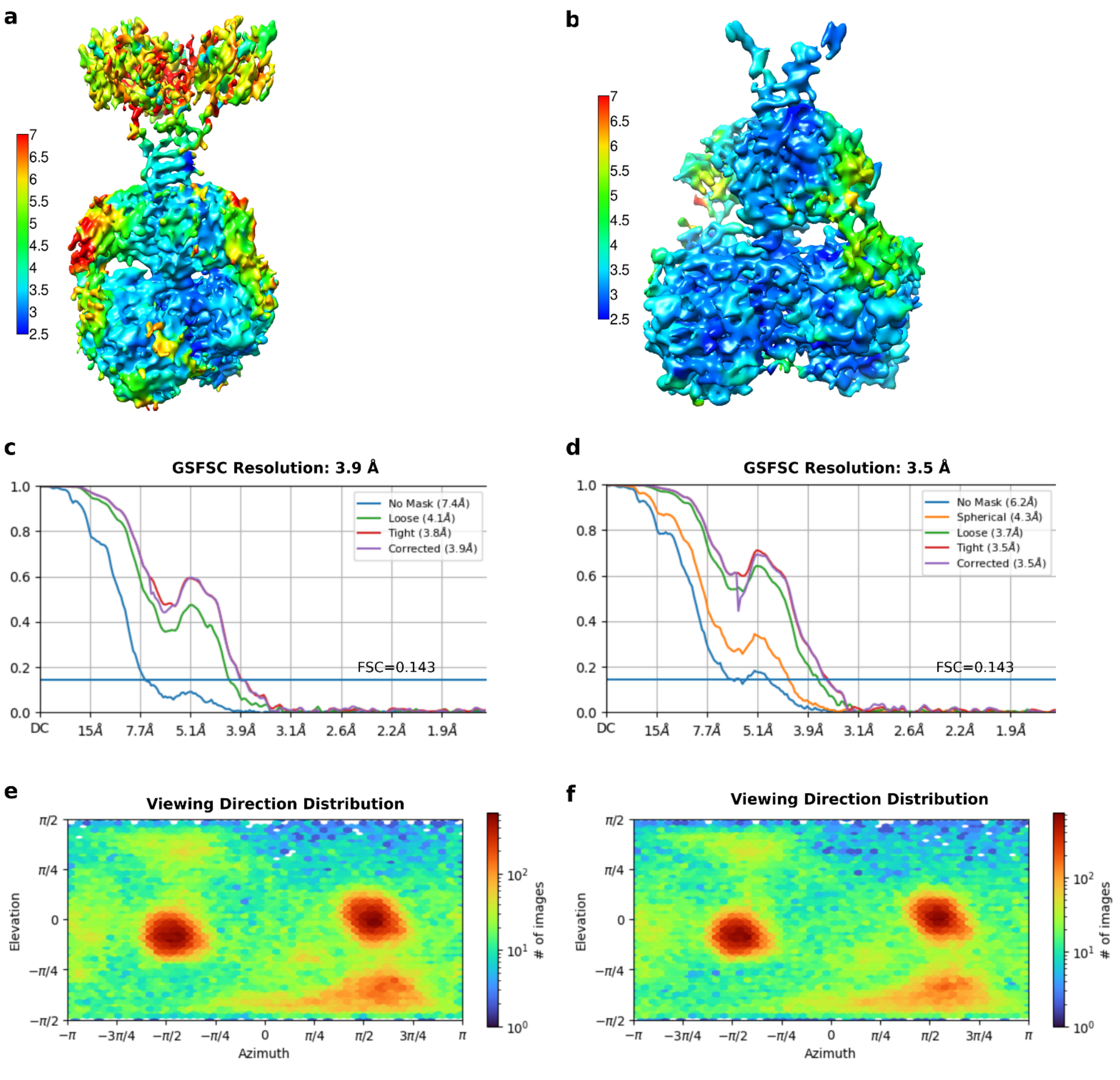

The sharpened cryo-EM densities of the Pfr state of (**a**) full-length and (**b**) PSM, colored based on local resolution produced using local resolution estimation in cryoSPARC. The Fourier shell correlation for the cryo EM maps of (**c**) full-length and (**d**) PSM are also shown. The resolution of map corresponds to FSC 0.143. Angular distribution calculated in cryoSPARC for particle projections of (**e**) full-length and (**f**) PSM. The heat maps show the number of particles for each viewing angle.

Supplementary Figure 8: Representative micrographs and selected 2D class averages of the Pr and Pfr state.

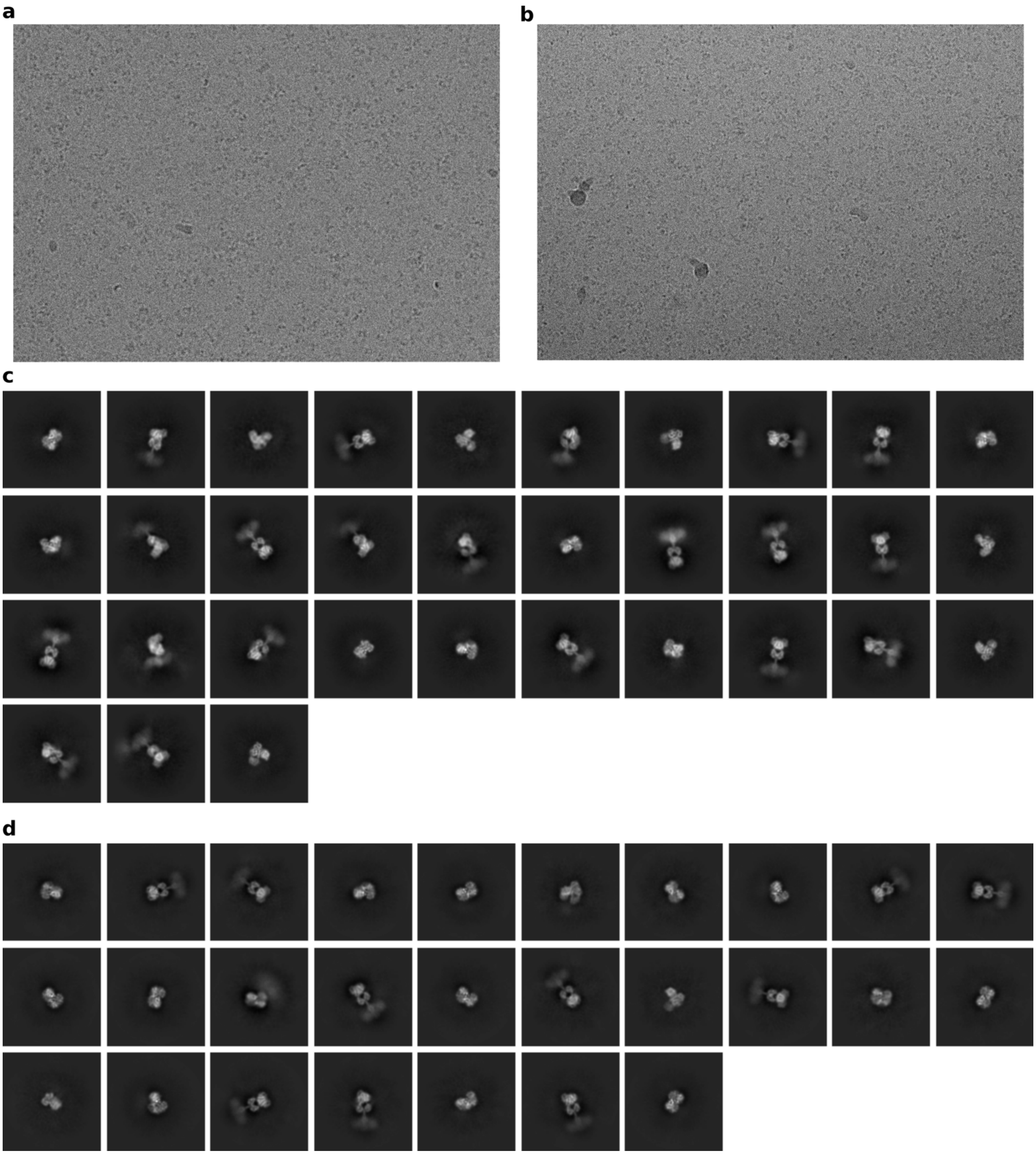

Representative micrographs collected from the grids illuminated with far-red (**a**) or red light (**b**). (**c**) The selected 2D class averages used for *ab initio* reconstruction step of the Pr state shown in Supplementary Fig. 2. (**d**) The selected 2D class averages after the second round of Topaz picking step of the Pfr state shown in Supplementary Fig. 4.

Supplementary references

1. E. Multamäki, R. Nanekar, D. Morozov, T. Lievonen, D. Golonka, W. Y. Wahlgren, B. Stucki-Buchli, J. Rossi, V. P. Hytönen, S. Westenhoff, J. A. Ihalainen, A. Möglich, H. Takala, Comparative analysis of two paradigm bacteriophytochromes reveals opposite functionalities in two-component signaling. *Nature Communications 2021 12:1*. **12**, 1–14 (2021).
